# Vegetal diamine oxidase alleviates histamine-induced contraction of colonic muscles

**DOI:** 10.1038/s41598-020-78134-3

**Published:** 2020-12-09

**Authors:** Armelle Tchoumi Neree, Rodolphe Soret, Lucia Marcocci, Paola Pietrangeli, Nicolas Pilon, Mircea Alexandru Mateescu

**Affiliations:** 1 Department of Chemistry, Research Chair on Enteric Dysfunctions “Allerdys”, University of Quebec at Montreal, Montreal, QC H3C 3P8 Canada; 2 Department of Biological Sciences, Research Chair on Rare Genetic Diseases, University of Quebec at Montreal, Montreal, QC H2X 3Y7 Canada; 3 Centre d’Excellence en Recherche sur les Maladies Orphelines - Fondation Courtois (CERMO-FC), University of Quebec at Montreal, Montreal, QC H2X 3Y7 Canada; 4 Department of Biochemical Sciences “A. Rossi Fanelli”, Sapienza University of Rome, 00185 Rome, Italy; 5 Department of Pediatrics, University of Montreal, Montreal, QC H3T 1C5 Canada

**Keywords:** Gastroenterology, Gastrointestinal diseases, Biochemistry

## Abstract

Excess of histamine in gut lumen generates a pronounced gastrointestinal discomfort, which may include diarrhea and peristalsis dysfunctions. Deleterious effects of histamine can be alleviated with antihistamine drugs targeting histamine receptors. However, many antihistamine agents come with various undesirable side effects. Vegetal diamine oxidase (vDAO) might be a relevant alternative owing to its histaminase activity. Mammalian intestinal mucosa contains an endogenous DAO, yet possessing lower activity compared to that of vDAO preparation. Moreover, in several pathological conditions such as inflammatory bowel disease and irritable bowel syndrome, this endogenous DAO enzyme can be lost or inactivated. Here, we tested the therapeutic potential of vDAO by focusing on the well-known effect of histamine on gut motility. Using ex vivo and in vitro assays, we found that vDAO is more potent than commercial anti-histamine drugs at inhibiting histamine-induced contraction of murine distal colon muscles. We also identified pyridoxal 5′-phosphate (the biologically active form of vitamin B6) as an effective enhancer of vDAO antispasmodic activity. Furthermore, we discovered that rectally administered vDAO can be retained on gut mucosa and remain active. These observations make administration of vDAO in the gut lumen a valid alternative treatment for histamine-induced intestinal dysfunctions.

## Introduction

Histamine is a friend and foe biogenic amine widely distributed and involved in major biological processes (e.g., neurotransmission, activity of smooth muscles and immune responses) through the activation of one or more of the four specific histamine receptors^1,2^. Histamine of endogenous or exogenous (food) origin may trigger drastic allergic phenomena. It can act as vasodilator/hypotensive agent^3^, may dangerously increase vascular permeability^4,5^, induce cardiorenal damages and arrhythmia^6,7^, and trigger death by anaphylaxis^8^.

Histamine, like other biogenic amines, is present at high level in fermented foods (cheese, sauerkraut, sausages), scombroid fish products (sardines, anchovies) and vegetables such as spinach and tomato^9–12^. Food histamine is not an allergen per se but, when present at high concentrations, it is the causative agent of food histaminosis^13–15^, related pseudo-allergies^16,17^ and inflammatory conditions^18^. The second main exogenous source of histamine consists of histamine-producing enterobacteria contained in the commensal microbiota^19,20^.

The four histamine receptors (H1R-H4R) are present on the surface of various immune cells (*e.g*., thrombocytes, neutrophils, eosinophils, mast cells, macrophages, lymphocytes T and B) as well as on endothelial, epithelial and muscular cells. In the bowel, histamine signaling is notably involved in the recruitment of immune cells^21,22^ and stimulation of smooth muscle contractility^23–25^, the latter being mediated at least in part by induction of calcium release from the endoplasmic reticulum into the cytosol^26^.

In situ concentration of histamine is controlled by the activity of catabolic enzymes such as histamine-N-methyl transferase and some copper/topaquinone-containing amine oxidases (Cu-AOs)^27^. The Cu-AOs, which are present in epithelial cells of various organs (*e.g.* intestine, kidney and placenta) are able to catalyze the oxidative deamination of the amino group of endogenous biogenic amines (*e.g.*, putrescine, cadaverine, spermidine, spermine and histamine) to the corresponding aldehyde, consuming oxygen with the concomitant release of stoichiometric amounts of ammonia and hydrogen peroxide. In particular, histamine is metabolized as follows:

$${\text{Histamine}} + {\text{O}}_{2} + {\text{H}}_{2} {\text{O}} \to {\text{Imidazole - 4 - acetaldehyde}} + {\text{H}}_{{2}} {\text{O}}_{{2}} + {\text{NH}}_{3}$$

In gut lumen, histamine is mainly catabolized by intestinal diamine oxidase (iDAO). However, iDAO levels are often decreased by certain drugs, alcohol intake and intestinal dysfunctions (*e.g.*, inflammatory bowel disease and irritable bowel syndrome), with the result that amounts of histamine largely exceed the capacity of iDAO to decompose it^17,28^.

Excess of histamine in gut lumen may trigger diarrhea, abdominal pain or constipation by increasing neurosecretory functions and muscle contractility^29,30^. Antihistamine agents targeting histamine receptors may help to alleviate histamine-induced enteric dysfunctions. Yet, such histamine receptor antagonists may also cause side effects such as rash, dry mouth, urinary retention, drowsiness and delirium^31,32^. As alternative to these synthetic antihistamine agents, we and others have previously proposed to treat histamine-related dysfunctions with vegetal diamine oxidase (vDAO) extracted from white or green pea^33–36^. DAO of vegetal origin is believed to have a better acceptability and to be safer in terms of secondary effects, while exhibiting higher specific activity than commercially available DAO of animal origin (*i.e.*, pig kidney DAO; pkDAO)^37^.

Using colon muscle contractility as proxy, the main objective of the current study was to validate the idea of using vDAO purified from *Lathyrus sativus* as therapeutic agent against gastrointestinal histamine excess. Based on prior reports showing an activating physical association between pkDAO and the vitamin B6 derivative pyridoxal 5′-phosphate (PLP)^38,39^, we also investigated the potential role of PLP as an enhancer of vDAO effect. In addition, we explored the possibility of rectal administration of vDAO by enema.

## Results

### Activity of endogenous iDAO is lower in the colon

**Figure 1:**
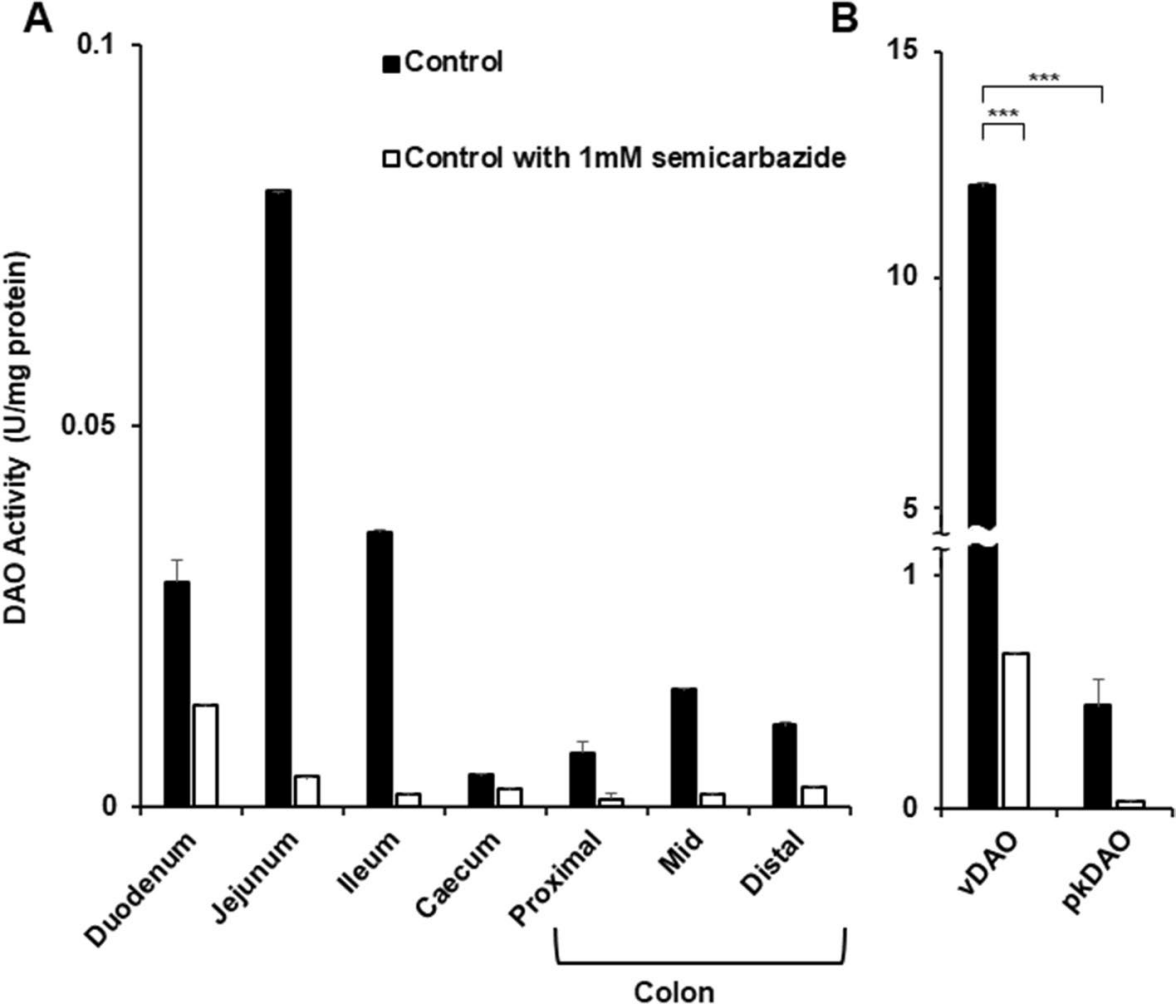
Assay of DAO activity from various sources by spectrofluorimetry. Specific activity expressed in units (U)/mg protein of DAO from (**A**) homogenates of various intestinal segments of mice and from (**B**) vegetal (*Lathyrus sativus*) and animal (pig kidney) sources in the absence (black) or presence (white) of semicarbazide (mean ± SD; n = 3; ** *P* < 0.01, *** *P* < 0.001; one-way ANOVA multiple comparisons).

The mammalian intestine is known to contain endogenous iDAO^40,41^, but with unclear distribution along the gastrointestinal tract. With the idea of selecting a region with low iDAO activity that we could use for ex vivo contractility assays, we first evaluated iDAO activity in different bowel segments of adult female mice. To this end, ability of iDAO to generate H_2_O_2_ upon oxidative deamination of putrescine was determined in extracts of duodenum, jejunum, ileum, cecum, proximal colon, mid colon and distal colon. This analysis revealed unequal distribution of iDAO activity along the gastrointestinal tract, with lowest activity in cecum and colon. Particularly, the distal colon displayed approximatively 4–10 times less of iDAO activity compared to the duodenum, ileum and jejunum, respectively (Fig. 1A).

Importantly, incubation with semicarbazide (a Cu-AO-specific inhibitor that covalently binds topaquinone in the active site)^39^ markedly reduced the enzymatic activity by about 60–95% (Fig. 1A), thereby confirming that the measured intestinal enzyme activity was due to a Cu-AO and not to another putrescine degrading enzyme. Using the same approach, we also evaluated the specific activity of partially purified vDAO and found it to be approximatively 30 times higher than that of commercial pkDAO (Fig. 1B). Based on these results we reasoned that the distal colon would best fit our needs. Moreover, these analyses confirmed the potency of vDAO over commercial pkDAO and iDAO.

### vDAO is a potent inhibitor of histamine-induced colon muscle contractions ex vivo

**Figure 2:**
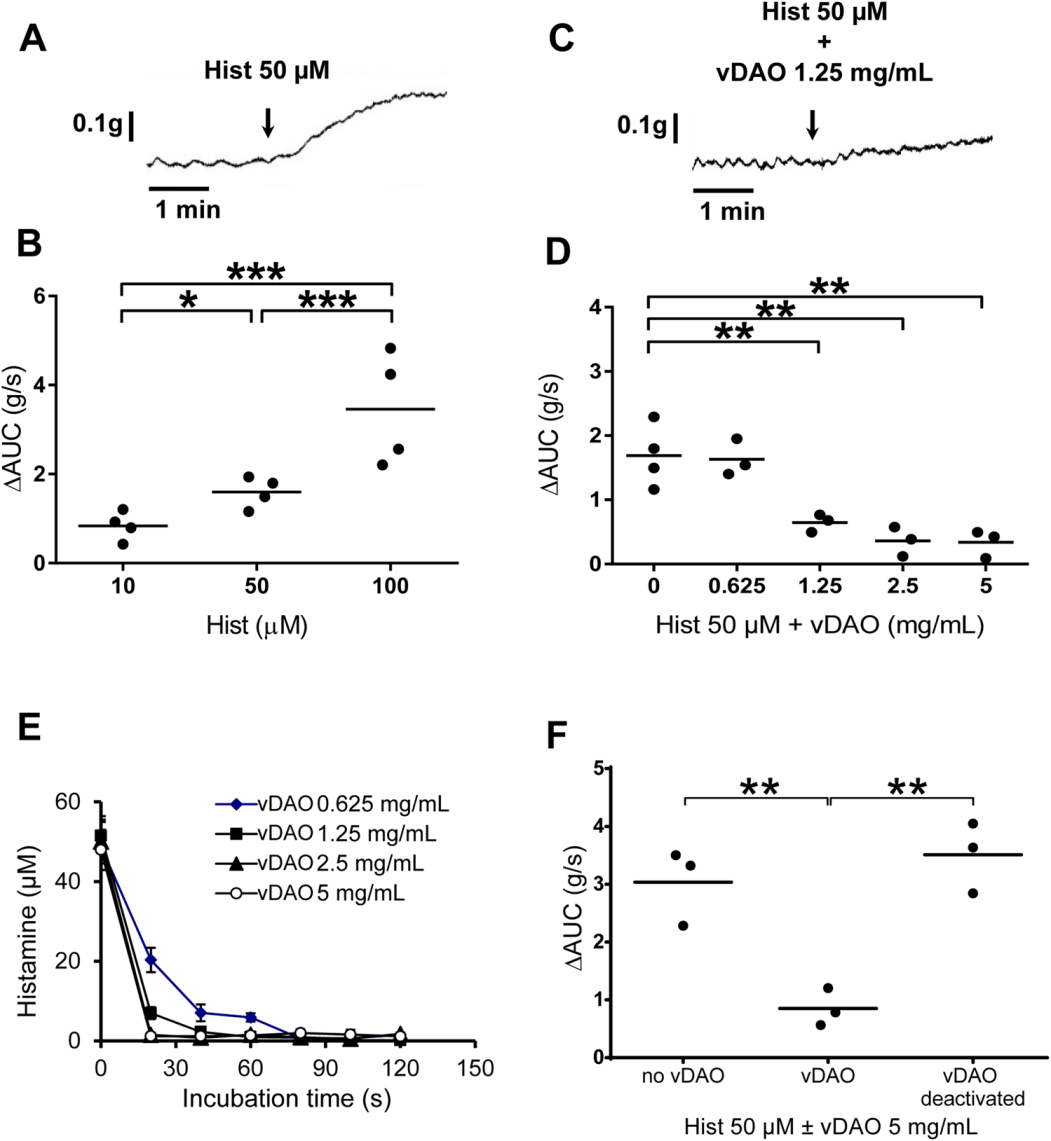
Time course of histamine-triggered distal colon muscle contraction in absence or in presence of vDAO ex vivo. Upper panels are representative contractility curves induced by 50 µM histamine (**A**) alone or (**C**) in presence of vDAO 1.25 mg/mL. Middle panels are quantitative analyses of contractility (expressed in g/s) corresponding to the difference in the area under the curve (ΔAUC) after and before addition of various concentrations of (**B**) histamine or (**D**) vDAO and 50 µM histamine. Lower panels are control experiments showing that oxidative deamination of histamine is slower at lowest concentration of vDAO (**E**), and that the vDAO preparation has no impact on histamine-induced contractions when inactivated with the specific Cu-AO inhibitor (topaquinone-binding) semicarbazide (**F**) (n = 3–4; * *P* < 0.05, ** *P* < 0.01, *** *P* < 0.001; one-way ANOVA multiple comparisons).

To evaluate vDAO’s impact on histamine-induced smooth muscle contractions, strips of distal colon muscles were prepared and attached to a force transducer in an organ bath. Using this ex vivo system, we first performed a dose–response assay with histamine only (at concentrations between 10 and 100 µM) which revealed that a concentration of 50 µM was sufficient to generate a robust spasmodic response (Fig. 2A,B). Using this concentration of histamine to induce muscle contractions, we then evaluated the effect of increasing concentrations of vDAO. At a low concentration of vDAO (0.625 mg solid/mL, about 2.5 µM), no significant response was observed in comparison to histamine only. But, at higher concentrations of 1.25, 2.5 and 5 mg solid/mL (about 5, 10 and 20 µM) vDAO, we observed a significant decrease of muscle contractions that appeared to reach a plateau at highest doses (Fig. 2C,D). These results are in line with the kinetics of histamine elimination by vDAO seen in vitro, where oxidative deamination of histamine was similarly quicker with 1.25 and 2.5 mg solid/mL of vDAO than with 0.625 mg solid/mL of vDAO (Fig. 2E). The apparent lack of ex vivo effect at lowest vDAO concentration is thus likely due to a limited capacity of vDAO to eliminate histamine before this small molecule (111 Da) can freely penetrate the muscles and presumably become somehow protected from the action of the much larger vDAO enzyme homodimer (150 000 Da^42^). It is also noteworthy that semicarbazide-inactivated vDAO had no impact on histamine-induced muscle contractions (Fig. 2F), ruling out the possibility that our vDAO preparation might have contained another substance with anti-histamine activity. Moreover, the muscle relaxing activity of vDAO seems to be specific for histamine-induced contractions as vDAO had no impact on L-NAME-provoked^43^ muscle contractions (Fig. S1).

**Figure 3:**
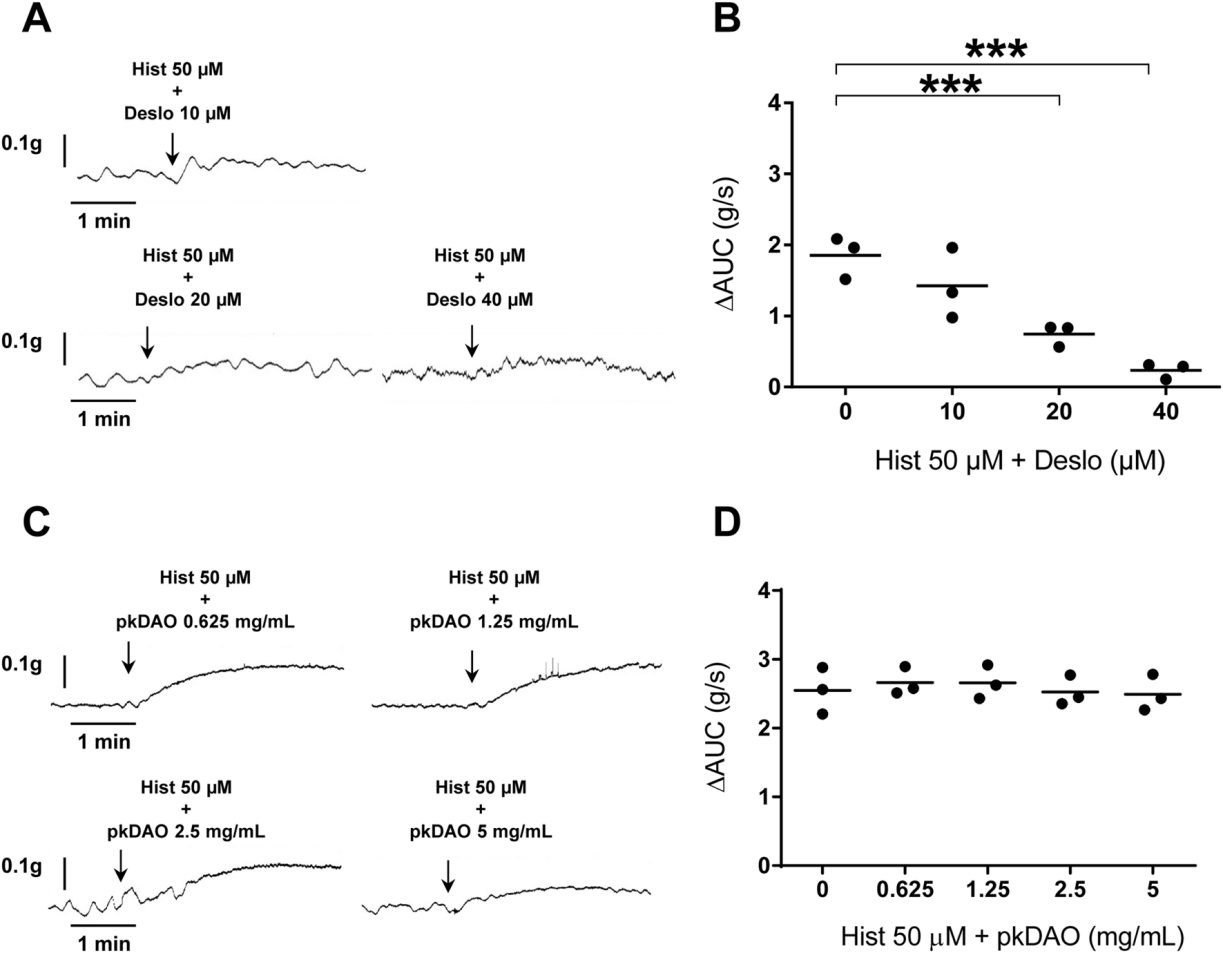
Effect of commercial antihistamine agents on histamine-triggered distal colon muscle contraction ex vivo. The effect of desloratadine (Deslo) antihistamine drug on the ex vivo histamine-induced contractile response (Hist 50 µM, left side) and the related quantitative analysis (right side) in the presence of (**A**,**B**) desloratadine or of (**C**,**D**) pig kidney (pk) DAO at various concentrations (n = 3; ** *P* < 0.01, *** *P* < 0.001; one-way ANOVA multiple comparisons).

Using the same experimental system, we then decided to compare vDAO’s impact to that of the well-known H1 receptor antagonist desloratadine. Starting with a concentration of 10 µM, desloratadine inhibited histamine-induced contractility in a concentration-dependent manner, with an almost complete inhibition at the highest tested concentration of 40 µM desloratadine (Fig. 3A,B). Yet, when compared to the inhibitory effect of vDAO, desloratadine appeared about four times less efficient based on the fact that results with 1.25 and 2.5 mg solid/mL of vDAO (about 5 and 10 µM, respectively) (Fig. 2D) were similar to results with 20 and 40 µM of desloratadine. Commercially available pkDAO had no effect under these experimental conditions, even when used at concentrations as high as 5 mg solid/mL (Fig. 3C,D). Collectively, these data suggest that vDAO is especially efficient at inhibiting histamine-induced colonic muscle contractions.

### H_2_O_2_ released during vDAO-mediated histamine deamination has no effect on muscle contractions

**Figure 4:**
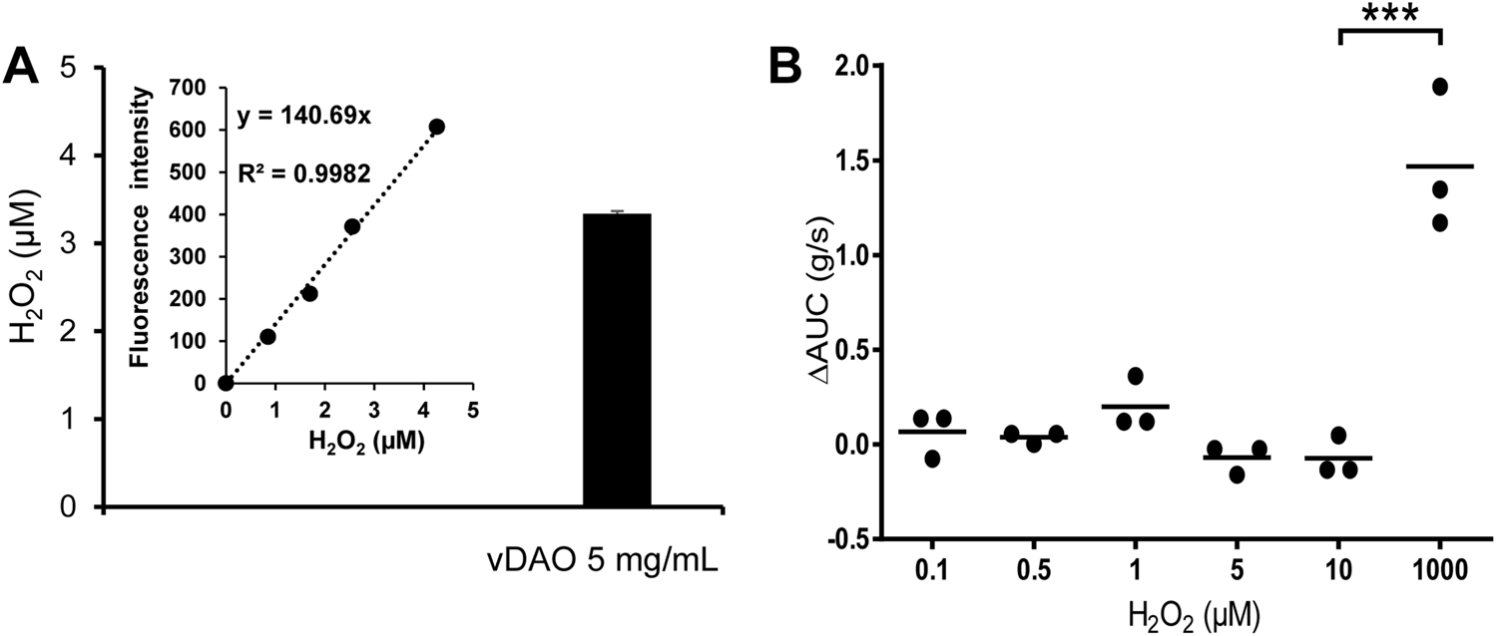
Effect of the H_2_O_2_ by-product of vDAO reaction on distal colon muscle contraction ex vivo. (**A**) Determination of H_2_O_2_ released in vitro by 5 mg solid/mL vDAO in presence of 50 µM histamine, using a H_2_O_2_ standard curve (inset in **A**). (**B**) Ex vivo effects of varying concentrations of H_2_O_2_ on muscle contraction (n = 3; *** *P* < 0.001; one-way ANOVA multiple comparisons).

One concern for using vDAO as antispasmodic agent is that accumulation of the H_2_O_2_ by-product could eventually lead smooth muscles to contract again. To evaluate this possibility, the amount of H_2_O_2_ released by vDAO during ex vivo inhibition of histamine-induced contraction was measured fluorimetrically in vitro using a standard curve of H_2_O_2_ (Fig. 4A, inset). We found that the oxidative deamination of 50 µM histamine at the highest tested concentration of 5 mg solid/mL vDAO produced 3.3 µM H_2_O_2_ (Fig. 4A). At this concentration, and even higher (up to 10 µM), H_2_O_2_ had no effect on smooth muscle contractions. A major increase in muscle contractions was detected only when we increased H_2_O_2_ concentration to 1 mM (about 300 times higher than the concentration produced by 5 mg solid/ml vDAO) (Fig. 4B).

### vDAO effect can be further increased by PLP

**Figure 5:**
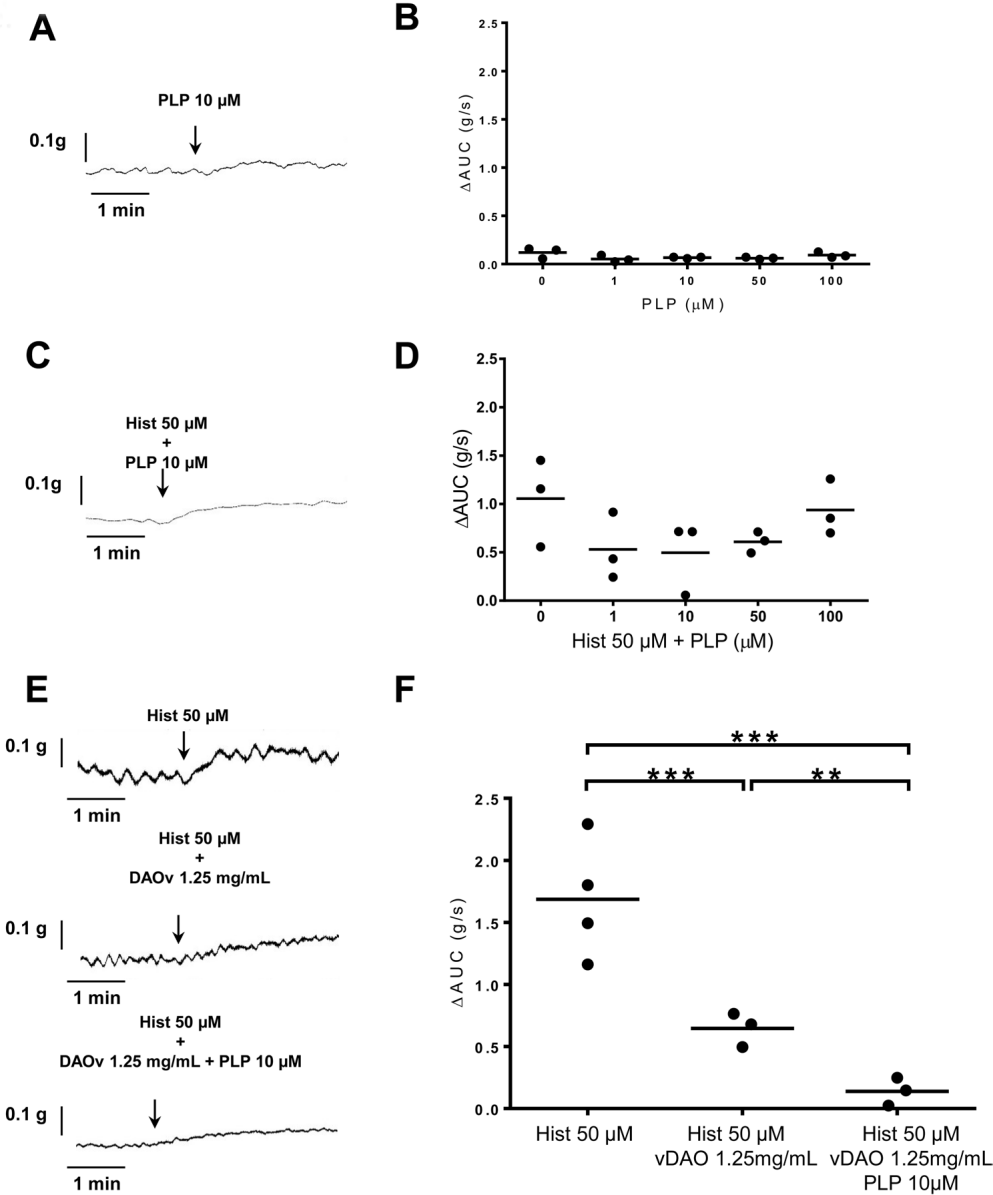
PLP enhances the antispasmodic effect of vDAO ex vivo. Representative contractility curves (left panels) and corresponding quantitative analyses (right panels) for (**A**,**B**) PLP alone, or in presence of (**C**,**D**) Histamine and of (**E**,**F**) Histamine and vDAO (n = 3; ** *P* < 0.01, *** *P* < 0.001; one-way ANOVA multiple comparisons).

Still using the same ex vivo system, we next tested the potential of PLP as vDAO modulator. PLP alone did not have an overt effect on basal (Fig. 5A,B) or on histamine-induced (Fig. 5C,D) contraction, regardless of PLP concentration used (up to 100 µM). We observed a small relatively uniform decrease in histamine-induced contraction with 1 to 50 µM of PLP (Fig. 5D) yet without reaching statistical significance, most likely because of small sample size. However, adding 10 µM of PLP to 1.25 mg/mL of vDAO was sufficient to elicit robust, statistically significant inhibition of histamine-induced contractile response in comparison to vDAO alone (Fig. 5E,F).

To more formally exclude the possibility that PLP might exert its effect by complexing histamine^44^, we compared the UV/VIS spectra of PLP ± histamine under the same experimental conditions used for ex vivo contractility assays (2 min of incubation at 37 °C in KH buffer in the organ bath). At the same concentrations used in these assays (and slightly higher; up to 200 µM), we found no differences between the PLP spectral pattern in the presence (Fig. S2A) or in absence (Fig. S2B) of 50 µM histamine. Robust PLP-histamine complexation appears to only occur in the millimolar range^44^, as we observed when we increased histamine concentration at 25 mM (about 500 times the concentration used in ex vivo assays). In these circumstances, we found that the peak of maximal absorbency for PLP (200 µM) is shifted from 390 to 410 nm (Fig. S2C).

**Figure 6:**
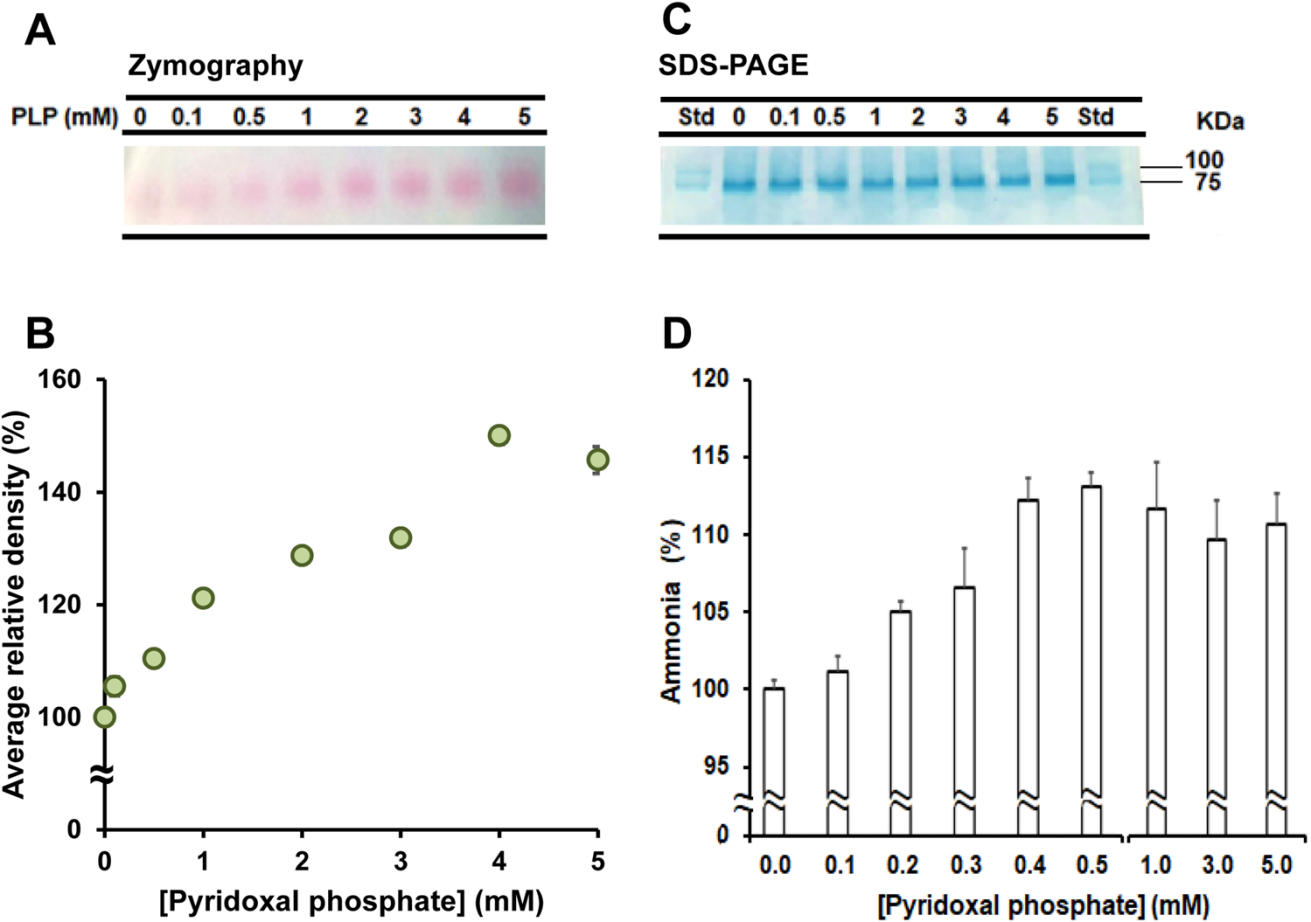
In vitro analysis of vDAO activity as a function of PLP concentration. (**A**) Representative zymography gel and (**B**) corresponding densitometry analysis of vDAO activity at different concentrations of PLP. (**C**) Coomassie staining of zymography gel indicating equal loading and appropriate molecular weight of vDAO monomer (75 kDa) according to Broad range SDS-PAGE molecular weight standards (std). (**D**) Ammonia concentration released during the vDAO activity in presence of various concentrations of PLP (values are mean ± SD; n = 3).

To further validate the enhancer role of PLP on vDAO, we measured vDAO activity in presence and absence of PLP using different in vitro approaches. Zymographic assays of 5 µg vDAO protein/well with increasing concentration of PLP ranging from 0.1 to 5 mM/well and staining with putrescine, AAP and DCHBS revealed an increase of vDAO activity as a function of PLP concentration. This increase appeared close to linear in presence of PLP concentrations ranging from 0.5 mM to 4 mM (Fig. 6A,B and Fig. S3). Coomassie blue staining confirmed that equal amounts of vDAO were loaded on gel (Fig. 6C and Fig. S3). Similar results were obtained when DAO activity was measured by evaluating the rate of ammonia production from putrescine oxidation (Fig. 6D). All these data suggest that the PLP-mediated increase of vDAO antispasmodic activity detected in our ex vivo contractility assays might be due to an interaction between PLP and vDAO rather than between PLP and histamine.

### Rectally-administered vDAO binds and remains active on colon mucosa

**Figure 7:**
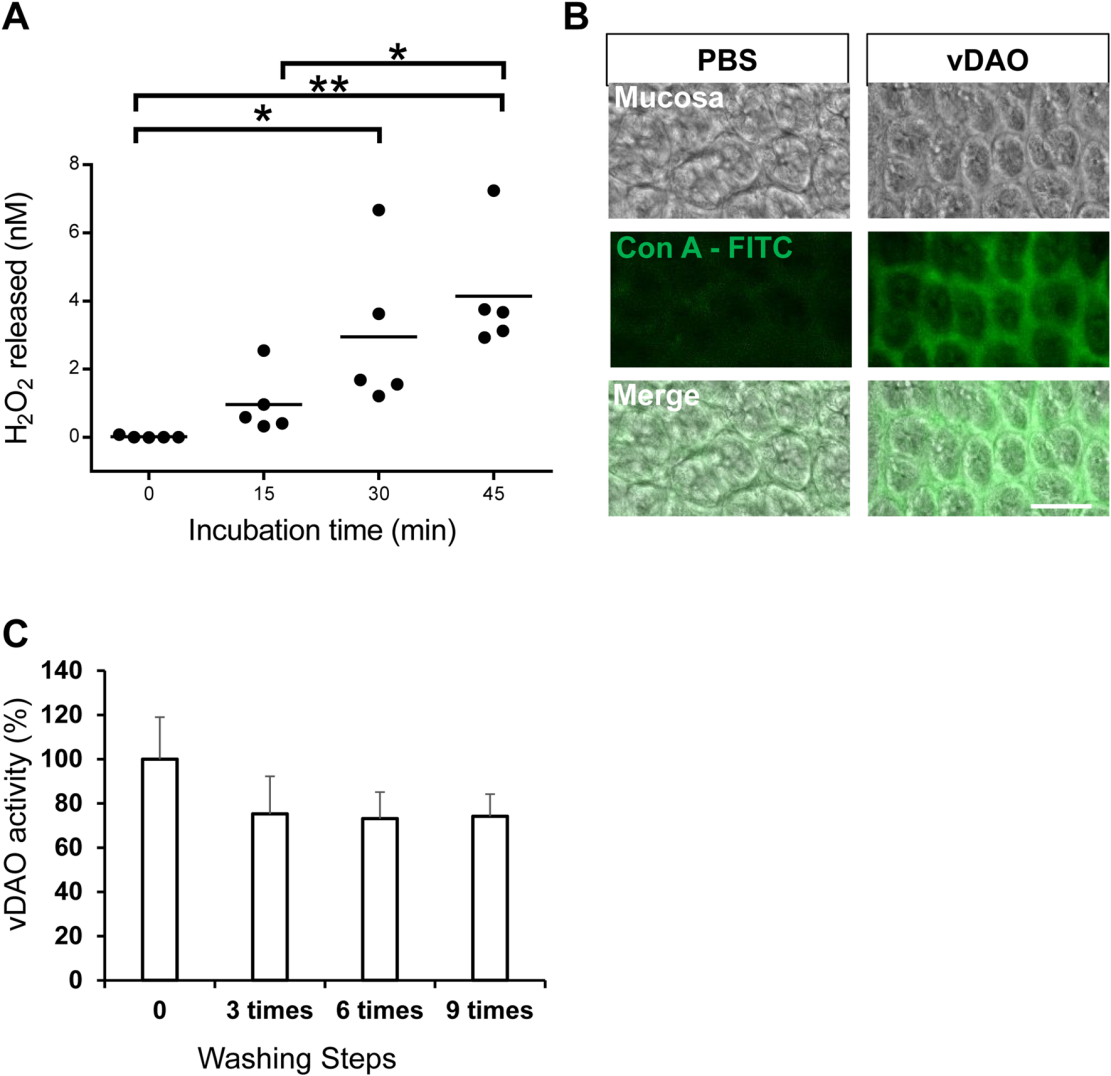
Retention of active vDAO on colon mucosa after rectal administration. (**A**) Analysis of vDAO-mediated release of H_2_O_2_ from colon mucosa of mice that previously received vDAO by enema. (**B**) Analysis of vDAO retention by staining with FITC-labeled Concanavalin A. (**C**) Retained vDAO remains active after repeated washes (mean ± SD; n = 3–5; * P < 0.05, ** P < 0.01; one-way ANOVA multiple comparisons).

With the idea of eventually using vDAO in the gut lumen to alleviate histamine-induced intestinal damage in humans, we evaluated whether vDAO can bind and remain active for a certain period of time on the intestinal mucosa in vivo. To this end, 100 µL of 1.25 mg solid/mL vDAO (in PBS, pH 7.0) was administered to adult female mice via rectal enemas. This enema volume was first established as being sufficient to fill the distal colon with methylene blue enema (Fig. S4). Mice that received vDAO or PBS only (control) enemas were sacrificed 15 min after enema administration and their distal colon (2 cm from the rectum) was analyzed for vDAO activity and mucosal retention, after extensive washing. Based on amounts of H_2_O_2_ released upon exposure to 50 µM histamine, the rectally-administered vDAO remained active for at least 45 min after injection (Fig. 7A). Binding of vDAO to the colon mucosa was confirmed by fluorescent staining of distal colon samples with FITC-conjugated concanavalin A, which is known to bind the carbohydrate moiety of Cu-AOs^45^ (Fig. 7B). Of note, retention of active vDAO on the colon mucosa also appeared stable, remaining the same regardless of the number of washing steps. Compared to unwashed freshly-dissected tissues for which measured vDAO activity was set at 100%, vDAO activity remained constant at 80% after 3, 6 and 9 successive washes (Fig. 7C). Altogether, these observations instill confidence for considering vDAO as therapeutic agent in the gut lumen.

## Discussion

Excess of histamine in the bowel can generate various gastrointestinal dysfunctions including motility problems. The current study strengthens the idea of using vDAO purified from *Lathyrus sativus* as therapeutic agent against such histamine-induced problems, and most especially in combination with the PLP enhancer. In addition, our data further suggest that the rectum could be a relevant and efficient route of administration.

Our in vivo and ex vivo data fit well with several previous studies. Our finding of unequal distribution of iDAO along the murine intestinal tract with lowest activity in the colon (Fig. 1A) is in agreement with studies reporting low amine oxidase activity in rat distal colon^46,47^. Sensitivity to semicarbazide (Fig. 1A) also confirmed that murine iDAO is a Cu-AO with topaquinone in the active site like pkDAO^48–50^. Based on our own in vivo observations and the existing literature, we thus selected the distal colon for our ex vivo assays of modulation of histamine-induced contractions. Histamine is well known to have a spasmodic effect on bowel smooth muscles, even at low concentration (1 µM)^51^. This contractility effect is mediated, at least in part, through H1 histamine receptors expressed in bowel smooth muscles^52^. Therefore, although we used a relatively high concentration of histamine (50 µM), the ability of desloratadine (an H1 antagonist) to robustly inhibit histamine-induced contraction of colon smooth muscles was nonetheless expected. What was more surprising is the discovery that vDAO has a markedly lower IC50 value as antihistamine agent (*i.e.*, concentration required to reduce histamine response by 50%) than desloratadine (5 µM for vDAO *vs* 20 µM for desloratadine).

We were further delighted to discover that addition of PLP could potentiate the vDAO effect, both ex vivo and in vitro. We turned to PLP in part because of early studies suggesting PLP could serve as prosthetic group for pkDAO^38,39^, although this concept was subsequently challenged following the identification of topaquinone as prosthetic group for many Cu-AOs^53^. Yet, the idea to use PLP (the biologically active form of vitamin B6) together with vDAO was also motivated by several studies reporting a link between vitamin B6 levels and bowel inflammation, although the mechanism underlying this relationship is unclear as both high and low levels of vitamin B6 were reported to have beneficial effects^54–56^. In the end, our data strongly suggest that exogenous PLP might have a dual therapeutic effect when used in conjunction with exogenous vDAO, most likely influencing both vDAO activity and endogenous PLP-dependent metabolic enzymes relevant for bowel inflammation and/or motility^57^. Indeed, while in vitro data clearly show that PLP can directly enhance vDAO activity in a dose-dependent manner (Fig. 6), this gain in activity is very modest at the lowest tested PLP concentration (100 µM) which is 10 times the concentration (10 µM) that we used with vDAO to elicit strong inhibition of histamine-induced contraction in ex vivo assays (Fig. 5E,F). This difference in magnitude, combined to the small decrease of histamine-induced contraction in presence of PLP alone (Fig. 5C,D), strongly suggests that PLP might activate some metabolic pathways in colon smooth muscles, independently of vDAO. One relevant pathway might be the transsulfuration pathway for which the by-product of cysteine degradation H_2_S is a smooth muscle relaxant^58,59^, while another one might be the degradation pathway of the pro-contractile sphingosine-1-phosphate^60,61^. We cannot also exclude the possibility that PLP might have another beneficial effect through formation of an inactive cyclic compound with histamine^62,63^. However, we note that this appears to be a relatively rare event in the micromolar range (involving only 7% of PLP at equimolar concentrations of 10 µM)^44^ and most likely the reason why it was not detected in our experimental conditions using histamine at 50 µM and PLP at 10–200 µM (Fig. S2A).

The ability of the colon mucosa to retain rectally administered vDAO in an active conformation is especially meaningful. The retention of vDAO might be explained by oxidative deamination of lysine ε-amino residues on proteins from the surface of the mucosa. This hypothesis is supported by a previous study suggesting a role for serum Cu-AO in post-translational modifications of proteins^64^ through recognition and oxidation of ε-amino side-chain of lysine (owing to structural similarity with lysine’s degradation product and natural Cu-AO target cadaverine). Cu-AOs including vDAO are thus supposedly able to oxidise the ε-amino side-chains of lysine which, once oxidised to an aldehyde, might then bind the own ε-amino groups of Cu-AO’s lysyl groups (a mechanism alike that of Schiff binding) as described for the aminohexyl (AH) chains on AH-Sepharose chromatographic material^65^. Importantly, retention of active vDAO on gut mucosa (Fig. 7A–C) suggests that a single oral^28,66,67^ or rectal (this work) administration would not be eliminated through intestinal transit (24 h) and might thus be efficient for a long time until renewal of the gut mucosa every 2–5 days^68,69^. The results of this study thus provide a strong impetus for clinical studies in humans.

## Materials and methods

### Materials

Aminoantipyrine (AAP), ammonia assay kit, bovine serum albumin (BSA), broad range SDS-PAGE molecular weight standards, concanavalin-A coupled with fluorophore fluorescein isothiocyanate (Con-A-FITC), desloratadine, 3,5-dichloro-2-hydroxybenzoic acid (DCHBS), histamine, hydrogen peroxide (H_2_O_2_), homovanillic acid (HVA), paraformaldehyde, horseradish peroxidase (HRP) type II, HRP type X, pig kidney diamine oxidase (pkDAO), protease inhibitor cocktail, putrescine, pyridoxal 5′-phosphate (PLP), semicarbazide and N-nitro-L-arginine methyl ester (L-NAME) were purchased from Sigma-Aldrich (Oakville, Ontario, Canada). Bradford reagent, acrylamide/bis-acrylamide solution (29:1) and Precision plus Protein Kaleidoscope Standards were from Bio-Rad Laboratory (Mississauga, Ontario, Canada). All current chemicals were Reagent Grade and were used without further purification.

### Purification of vDAO

The vDAO enzyme was prepared from *Lathyrus sativus* seedlings as per our prior work^70^, by homogenizing *Lathyrus sativus* seedlings in 50 mM phosphate buffer (pH 5.5) containing 200 mM NaCl. Purified vDAO solutions were lyophilized and characterized as previously reported^71^. vDAO samples containing 0.64 ± 0.095 mg protein/mg solid (19.0 ± 7.92 U DAO/mg solid) were used for further experiments.

### Chemical deactivation of vDAO

A 5 mg solid/mL vDAO solution was incubated with 10 mM semicarbazide in Krebs–Henseleit buffer (KH), pH 7 at 37 °C for 6 h. Then, the solution was dialysed overnight in 4L of 50 mM phosphate buffer, pH 7.4 and stored frozen until experiments.

### Histamine oxidative decomposition by vDAO

Histamine (50 µM) was incubated at 37 °C in KH buffer in presence of various concentrations of vDAO. Control samples contained vDAO alone. At various intervals, 40 µL aliquots were withdrawn, added to 2 mL HCl 0.1 M and treated with ophthaldialdehyde under the conditions previously reported^71^. For each sample the fluorescence was read at λ_em_ = 450 nm upon λ_ex_ = 360 nm on a Perkin Elmer LS50B spectrometer and the histamine concentration was evaluated by referring to an established standard curve after subtraction of the fluorescence related to the control containing vDAO alone at the corresponding concentration.

### Animal housing and treatments

Experiments on mice were conducted following Canadian Council of Animal Care (CCAC) guidelines for the care and manipulation of animals used in medical research, with approval from the Institutional Animal Protection Committee [Comité Institutionnel de Protection des Animaux (CIPA) reference number #650]. Animals used in this study were FVB/N female mice of 3–4 months of age provided by Charles River (Quebec, Canada). These mice were housed at UQAM’s animal facility under a 12 h/12 h (light/dark) cycle and they were not starved before euthanasia (ad libitum access to water and standard chow pellets). Euthanasia was performed via CO_2_ inhalation, following anesthesia with isoflurane. Bowel tissues were then dissected for different experiments as described below.

### Preparation of intestine homogenates

Intestine from FVB/N mice were split in segments corresponding to duodenum, jejunum, ileum, caecum, proximal colon, mid colon and distal colon. Each fraction was homogenized in 10 volumes of cold Krebs–Henseleit buffer (KH) containing (in mM): 117 NaCl, 4.7 KCl, 1.2 MgCl_2_, 1.2 NaH_2_PO_4_, 25.0 NaHCO_3_, 2.5 CaCl_2_, and 11.0 glucose at pH 7, supplemented with 1% protease inhibitor cocktail with an ultrasonic liquid processor (Sonics vibra-cell, Sonics & Materials inc., Newtown, CT, USA) for 5 min (pulses of 15 s every 5 s) at 30 amplitude. The samples were dialysed at 4 °C in PBS 50 mM, pH 7.4, overnight and then centrifuged at 1000 g for 15 min. The supernatants were collected and store on ice until analysis of iDAO activity by fluorimetric assay. The protein concentrations were determined by Bradford method^72^ with bovine serum albumin as standard.

### Assay of DAO activity

#### Fluorimetric assay in terms of H_2_O_2_ generation

DAO catalytic activity was assayed spectrofluorimetrically by evaluating the generation of H_2_O_2_ upon the oxidative deamination of putrescine as previously reported^73^, with slight modifications. Briefly, samples at a final concentration of 0.015 mg protein/mL of intestinal homogenates, pkDAO or vDAO were added to KH buffer (final volume 150 µL) containing 8.37 mM HVA, 1 U/mg protein of HRP type X, 1 mM putrescine with or without 1 mM semicarbazide (for inhibition tests of copper–amine oxidases with topaquinone in the active site) and incubated at 37 ^o^ C for 30 min. A volume of 2.5 mL of NaOH 0.1 M was then added and the developed reaction was measured at 25 °C in a PerkinElmer LS45 fluorimeter (PerkinElmer, Waltham, MA, USA), using 315 nm (excitation) and 425 nm (emission) wavelengths. Samples incubated without the putrescine substrate were considered as blank. For each sample, the fluorescence value of the blank was subtracted from the measured value prior to report to a standard curve prepared from commercial H_2_O_2_. One enzyme unit was the amount of enzyme producing 1 µmole H_2_O_2_/min.

#### Spectrophotometric assay in terms of NH_3_ generation

Activity of vDAO in presence of PLP or not was also evaluated at 340 nm in terms of rate of NH_3_ formation during the oxidation of 3 mM putrescine in KH buffer at 37 °C, using an NH_3_ assay kit as per our prior work^66^. One enzyme unit was the amount of enzyme producing 1 μmole NH_3_/min.

#### Zymography assay

The zymographic assay of vDAO activity was performed as previously reported^74^ with slight modifications. vDAO samples (1 mg protein/mL) with or without PLP (at various concentrations) were diluted 1:1 with non-reducing SDS loading buffer containing 0.085 mM Tris–HCl at pH 6.8, 10% glycerol and 2% SDS. An amount of 5 µg of vDAO protein was loaded on a 10% SDS-PAGE resolving gel prepared from acrylamide/bisacrylamide solution containing entrapped HRP type II at a final concentration of 10 U/mL. Samples were run at 120 V for 1.5 h. The activity of vDAO was detected by treating the gel with a preheated (5 min at 37 °C) solution containing 30 mM putrescine, 1.25 mM AAP and 1.25 mM DCHBS for 30 min at 25 °C. The intensities of the active vDAO bands were quantified using the Image J software and normalized by subtracting the background signal from the gel (band-free area). To reveal the quantity of loaded protein, the zymography gel was destained by incubation with a methanol solution 50% v/v for 5 min and stained again with a Coomassie blue solution containing (40:10:50, v/v/v) methanol–acetic acid–water and 0.5% Coomassie Brilliant Blue G-250.

### Ex vivo effect of DAO and other agents on muscle contractility activated by histamine

Segments of mouse distal colon (2 cm from the anus) were cleaned of their luminal contents with oxygenated KH buffer (95% O_2_ and 5% CO_2_), and attached in the longitudinal direction in an organ bath (Harvard apparatus) filled with KH buffer at 37 °C. The isolated colon fragment was initially stretched with a preload of 1–2 g of tension. After 1 h of equilibration, the investigated agents (alone or in combination): histamine (10, 50 and 100 µM), desloratadine (10, 20 and 40 µM), PLP (1, 10, 50 and 100 µM), vDAO or pkDAO (0.625, 1.25, 2.5, and 5 mg solid/mL), H_2_O_2_ (0.1, 0.5, 1, 5, 10, and 1000 µM) and/or L-NAME (0.5 µM) were added to the organ bath and contractibility was measured up to 5 times. The contractile response of longitudinal muscle was continuously recorded with a myograph (model F-60, Narco-Biosystems, Houston, TX, U.S.), coupled to a Windows 7 computer equipped with the BIOPAC student Lab 4.0.2 (BIOPAC Systems Inc., Lorraine, QC, Canada). Contractile strength in g/s was calculated as the difference from baseline of the area under the curve (AUC), and data are expressed in ΔAUC (corresponding to the difference between the AUC measured 2 min after addition of tested bioactive agents minus the AUC measured 2 min before the treatment).

### Investigation of Pyridoxal-5-phosphate (PLP) interaction with histamine

To better understand the decrease of contractility, the behaviour of PLP (10–200 µM) in presence of histamine (50 µM or 25 mM final concentration) in KH buffer (pH 7 at 25 °C) was followed in vitro by UV–VIS spectrophotometry from 220 to 600 nm. Solutions of 50 µM histamine (final concentration), with various concentrations of PLP up to 100 µM with and without vDAO (1.25 mg/mL) were also prepared in the same buffer and used in ex vivo colon contractility assays as described above.

### In vivo analysis of rectally administered vDAO

vDAO was administered to anesthetized mice, using colonic enemas. To this end, the head of a 20-gauge cannula (Fine Science Tools, Vancouver, Canada) was introduced in the mouse rectum with vaseline and a volume of 100 µL vDAO was administered. This volume was determined in preliminary experiments in 1-month-old mice (n = 3) with methylene blue (10% diluted in PBS, pH 7) enemas. It was found that 100 µL was sufficient to fill the distal colon. Consequently, 100 µL of vDAO (1.25 mg solid/mL) or PBS (control) were injected by enema and, after 15 min, animals were euthanized by CO_2_ inhalation. For vDAO activity assays, the distal colon was then dissected, repeatedly washed in PBS (cycles of 3, 6 and 9 washes), homogenized and assayed for enzymatic activity as described above. For vDAO localization assays, the distal colon was dissected, rinsed with PBS and fixed in paraformaldehyde (4% in PBS, pH 7) for 24 h. The tissue was then opened longitudinally and mucosa side was incubated for 30 min with 35 µg/mL Con-A-FITC (Concanavalin-A coupled with fluorescein isothiocyanate). Stained tissues were finally washed in PBS and mounted on microscope slides for imaging with a Nikon Eclipse Ti inverted fluorescence microscope (Nikon Instruments Inc., Melville, NY, U.S.A.). Image acquisition was performed with a Scion Model CFW 1612C camera (Scion corporation, Frederick, MD, U.S.A.), using the Scion Image software.

### Statistics

All experiments used a minimum of three replicates. Where relevant, data are expressed as the mean ± SEM. Statistical tests were performed with the GraphPad software, using either 1-way ANOVA (for comparison between 3 groups) or 2-tailed Student’s *t*-test (for comparison between 2 groups). Differences were deemed statistically significant when the associated *P*-value was lower than 0.05.

## Supplementary information

Supplementary Information.

## Data Availability

All materials, data and associated protocols will be made promptly available to readers.

## Abbreviations

AAP: 4-Aminoantipyrine
deslo: Desloratadine
DAO: Diamine oxidase
DCHBS: 3,5-Dichloro-2-hydroxybenzoic acid
Hist: Histamine
HR: Histamine receptor
HDC: Histidine decarboxylase
HVA: Homovanillic acid
HRP: Horseradish peroxidase
IBD: Inflammatory bowel diseases
iDAO: Intestinal diamine oxidase
IBS: Irritable bowel syndrome
MW: Molecular weight
pkDAO: Pig kidney diamine oxidase
PLP: Pyridoxal 5′-phosphate
SDS-PAGE: Sodium dodecyl sulfate polyacrylamide gel electrophoresis
vDAO: Vegetal diamine oxidase
