# Supplementary material for: Vegetal diamine oxidase alleviates histamine-induced contraction of colonic muscles
Source: Sci Rep. 2020 Dec 9;10:21563. doi: 10.1038/s41598-020-78134-3 (PMC7726047; doi:10.1038/s41598-020-78134-3)
Supplement: Supplementary file 1 — Supplementary Information. [file 41598_2020_78134_MOESM1_ESM.docx]

**VEGETAL DIAMINE OXIDASE ALLEVIATES HISTAMINE-INDUCED CONTRACTION OF COLONIC MUSCLE*S***

Armelle Tchoumi Neree^1,3,#^, Rodolphe Soret^2,3,#^, Lucia Marcocci^4^, Paola Pietrangeli^4^, Nicolas Pilon^2,3,5*^, Mircea Alexandru Mateescu^1,3^*

^1^ Department of Chemistry, Research Chair on Enteric Dysfunctions "Allerdys", University of Quebec at Montreal, Montreal (QC) H3C 3P8, Canada

^2^ Department of Biological Sciences, Research Chair in Rare Genetic Diseases, University of Quebec at Montreal, Montreal (QC) H2X 3Y7, Canada

^3^ Centre d'Excellence en Recherche sur les Maladies Orphelines - Fondation Courtois (CERMO-FC), University of Quebec at Montreal, Montreal (QC) H2X 3Y7, Canada

^4^ Department of Biochemical Sciences “A. Rossi Fanelli”, Sapienza University of Rome, Rome 00185, Italy

^5^ Department of Pediatrics, University of Montreal, Montreal (QC) H3T 1C5, Canada.

^#^ Contributed equally

**Corresponding Authors**:

*Mircea Alexandru Mateescu *Nicolas Pilon

Department of Chemistry Department of Biological Sciences

University of Quebec at Montreal University of Quebec at Montreal

Montreal, Quebec H3C 3P8, Canada Montreal, Quebec H2X 3Y7, Canada

Phone number: +1(514) 987-3000 x4319 +1(514) 987-3000 x3342

E-mail: [mateescu.m-alexandru@uqam.ca](about:blank) pilon.nicolas@uqam.ca

**SUPPLEMENTARY INFORMATION (4 figures)**

**Figure S1. vDAO has no impact on L-NAME-provoked colon muscle contractions *ex vivo*. Figure S2. UV-Vis absorption spectra of PLP with and without histamine.**

**Figure S3.** **Uncropped gels of Fig.6**

**Figure S4.** **Evaluation of enema volume required to fill the distal colon.**

**Fig. S1. vDAO has no impact on L-NAME-provoked colon muscle contractions *ex vivo*.** Quantitative analyses of contractility (expressed in g/s) corresponding to the difference in the area under the curve (ΔAUC) after and before addition of L-NAME alone or in combination to vDAO.


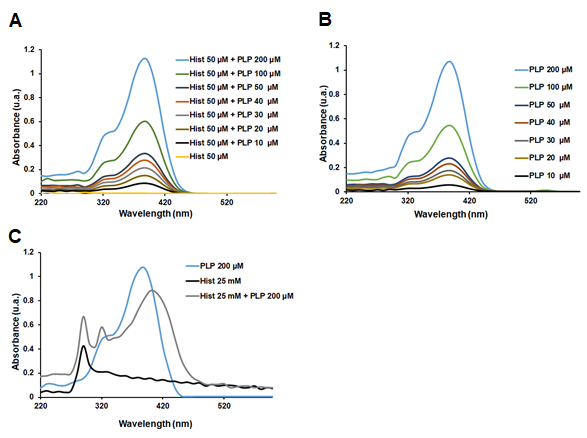


**Fig. S2: UV-Vis absorption spectra of PLP with and without histamine.** Absorption spectra of (A) 50 µM histamine (Hist) and PLP at concentrations used in *ex vivo* assays, (B) histamine-free PLP (as negative control) and (C) higher histamine (25 mM) and PLP (200 µM) concentrations (as positive control).

**
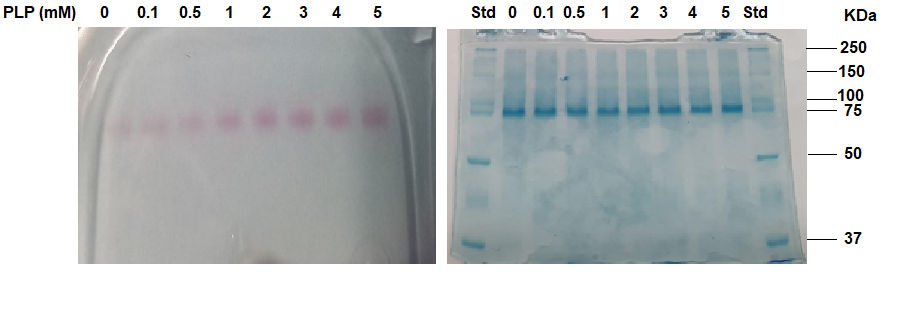
**

**Zymography**

**SDS-PAGE**

**Fig. S3.** **Uncropped gels of Fig.6**

**Fig. S4.** **Evaluation of enema volume required to fill the distal colon.** A volume of 100 µL of methylene blue was rectally-administered *in vivo* to FVB female mice , which were then sacrificed to evaluate methylene blue distribution in the distal colon (representative of n=3 evaluations).
